# Upper Arm Central Venous Port Implantation: A 6-Year Single Institutional Retrospective Analysis and Pictorial Essay of Procedures for Insertion

**DOI:** 10.1371/journal.pone.0091335

**Published:** 2014-03-10

**Authors:** Masatoshi Shiono, Shin Takahashi, Yuichi Kakudo, Masanobu Takahashi, Hideki Shimodaira, Shunsuke Kato, Chikashi Ishioka

**Affiliations:** 1 Department of Clinical Oncology, Tohoku University Hospital, Tohoku University, Aoba-ku, Sendai, Japan; 2 Department of Clinical Oncology, Institute of Development, Aging, and Cancer, Tohoku University, Aoba-ku, Sendai, Japan; Yonsei University College of Medicine, Republic of Korea

## Abstract

**Background:**

The requirement of central venous (CV) port implantation is increasing with the increase in the number of cancer patients and advancement in chemotherapy. In our division, medical oncologists have implanted all CV ports to save time and consultation costs to other departments. Recently, upper arm implantation has become the first choice as a safe and comfortable method in our unit. Here we report our experience and discuss the procedure and its potential advantages.

**Methods:**

All CV port implantations (n = 599) performed in our unit from January 2006 to December 2011 were analyzed. Procedural success and complication rates between subclavian and upper arm groups were compared.

**Results:**

Both groups had similar patient characteristics. Upper arm CV port and subclavian implantations were equivalently successful and safe. Although we only retrospectively analyzed data from a single center, the upper arm group had a significantly lower overall postprocedural complication rate than the subclavian group. No pneumothorax risk, less risk of arterial puncture by ultrasound, feasibility of stopping potential arterial bleeding, and prevention of accidental arterial cannulation by targeting the characteristic solitary basilic vein were the identified advantages of upper arm CV port implantation. In addition to the aforementioned advantages, there is no risk of “pinch-off syndrome,” possibly less patient fear of manipulation, no scars on the neck and chest, easier accessibility, and compatibility with the “peripherally inserted central catheter” technique.

**Conclusions:**

Upper arm implantation may benefit clinicians and patients with respect to safety and comfort. We also introduce our methods for upper arm CV port implantation with the videos.

## Introduction

**Figure 1:**
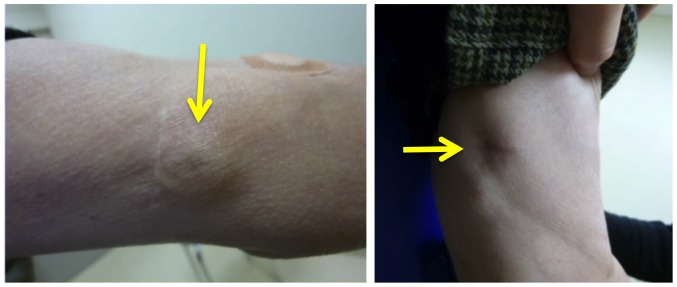
Appearance of the upper arms after CV port implantation. *Arrows* indicate implanted ports in the upper arm. It generally takes less effort to roll up a sleeve to provide access to the upper arm port than to completely remove a shirt for access to the subclavian port.

The number of cancer patients has been increasing worldwide due to progressive society aging. Rapid developments in outpatient cancer chemotherapy have exponentially increased the need for implantable central venous (CV) ports.[1] In 1993, forearm CV port implantation was reported to be a new optional procedure by surgeons at the Yale University School of Medicine;[2] however, venous access depended on visual confirmation of a distended median basilic vein. Alternatively, a small transverse skin incision was recommended, which is difficult for physicians who are not surgeons. Between 2006 and 2011, we implanted about 600 CV ports in the upper arm or upper chest subclavian regions of patients with advanced cancer who required chemotherapy. Here we introduce a relatively new practical method for CV port implantation in the upper arm (Fig. 1; described in the report and videos). Choice of upper arm and ultrasound guidance enable surgeons and other physicians such as medical oncologists to safely perform this procedure. Moreover, this technique can be applied to the “peripherally inserted central catheter” technique.

## Methods

### Inclusion and Exclusion Criteria, Patients, and Materials

For inclusion in this retrospective analysis, CV port implantations performed by physicians in the Division of Medical Oncology, Tohoku University Hospital, between January 2006 and December 2011 (n = 599) were investigated by chart review. Exclusion criterion was implantation in a site other than the subclavian area or the upper arm. Femoral implantation was chosen for only one case because of superior vena cava (SVC) syndrome. All patients were adults and diagnosed with recurrent or metastatic solid tumors. Thus, the indication for CV port implantation was continuous systemic chemotherapy. The distribution of patients according to primary cancer sites is shown in Table 1. Written informed consent was obtained from all patients before the procedure. All CV ports were implanted by two physicians (operator and assistant) from the same unit (Division of Medical Oncology, Tohoku University Hospital), regardless of subclavian or upper arm procedures. All CV ports were single lumen. The X-Port isp implantable port (Bard Access Systems, Inc., Salt Lake City, UT, USA) or SlimPort (Bard Access Systems) were used in the subclavian or upper arm procedures, respectively. Periprocedural antibiotic prophylaxis was administered in all cases. Ultrasound guidance for punctures and fluoroscopic guidance for catheter insertion were used in all cases. All adverse events were followed by each attending physician and recorded in the patient's medical chart upon occurrence. All observed adverse events are shown in Table 2.

**Table 1:** Characteristics of the Patients.

Characteristics	Implantation site		
	Subclavian (N = 342)	Upper arm (N = 257)	P Value
Male Sex - no. (%)	212 (62.0)	163 (63.4)	0.73
Median age (yr)	62	64	0.22
Malignancy by site^*^ - no. (%)			
Colorectal cancer	179 (52.3)	57 (22.2)	<0.001
Esophageal cancer	66 (19.3)	68 (26.5)	0.05
Gastric cancer	51 (14.9)	51 (19.8)	0.12
Others^†^	59 (17.3)	91 (35.4)	<0.001

**Table 2:** Proportion of Success and Complications.

Variable	Implantation site		
	Subclavian (N = 342)Upper arm (N = 257)		P Value
	*no. (%)*		
Procedural success			
Completion at the intended site^*^	340 (99.42)	254 (98.83)	0.66
Periprocedural complications			
Pneumothorax	3 (0.88)	0	0.26
Arterial puncture	2 (0.58)	0	0.51
Total	5 (1.46)	0	0.07
Postprocedural complications			
Infection†	14 (4.09)	8 (3.11)	0.66
Venous thrombosis	4 (1.17)	3 (1.17)	1.00
Catheter occlusion	6 (1.75)	0	0.04
Catheter fracture	3 (0.88)	0	0.26
Catheter malposition	3 (0.88)	0	0.26
Skin dehiscence	4 (1.17)	0	0.14
Leakage	1 (0.29)	1 (0.39)	1.00
Total	35 (10.23)	12 (4.67)	0.01

† Total parenteral nutrition was administered in all cases.

### Statistical Analysis

Fisher's exact test was used to compare patient characteristics and the rates of successful procedures and complications between the subclavian and upper arm groups. JMP software, version 10.0 (SAS Institute, Cary, NC, USA) was used for all statistical analyses. In all cases, two-sided P values of <0.05 were considered significant. All statistical analysis results were confirmed by an expert medical statistician.

### Ethics Statement

This is a retrospective observational analysis in which data sources were managed and analyzed using an anonymous patient code. The Tohoku University School of Medicine Institutional Review Board approved this study.

### Pictorial Description of Upper Arm CV Port Implantation

#### Preparation

**Figure 2:**
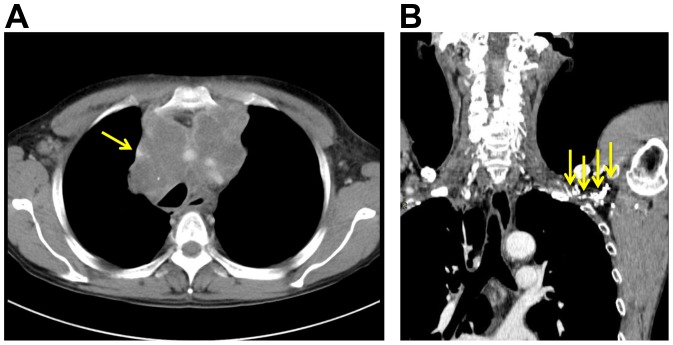
Examples of anticipated preoperative difficulties with the procedure confirmed on CT scan images. (**A**) **SVC syndrome.** *Arrow* indicates the excluded SVC by a tumor. (**B**) **Tortuous collateral blood circulation.** *Arrows* indicate the contrast-enhanced tortuous collateral blood circulation attributable to a modification caused by surgery, radiation, or spontaneous occlusion.

To reduce the difficulty in introducing a subsequent guide wire or catheter after puncture, we recommend checking the vessel course pattern on a contrast-enhanced computed tomographic scan image before determining the operative side. If there is stenosis, occlusion, or thrombosis in the vessel from the upper arm to the superior vena cava (SVC), the corresponding side must be avoided (Fig. 2A). Past history of radiation or surgery in the head and neck or chest on the same side should also be considered for collateral vessel development possibility (Fig. 2B). When there are no such risks, the opposite side of the dominant arm is the first choice. A preoperative peripheral intravenous infusion is preferable to circumvent the vessel collapse due to dehydration.

#### Prescan

**Figure 3:**
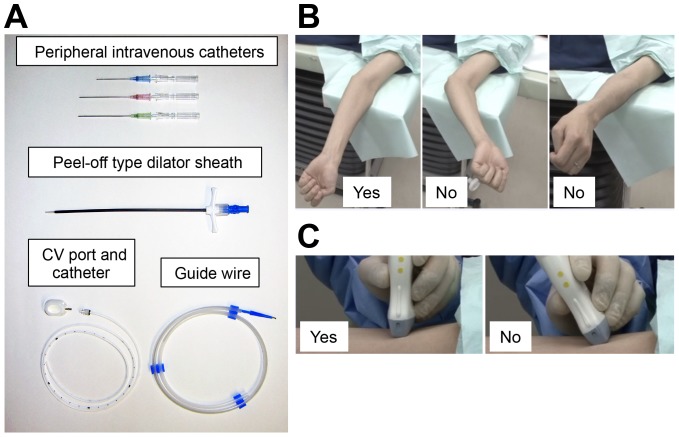
Preparation. (**A**) **Minimum specific materials for the procedure.** In this kit, a CV port/catheter, a dilator sheath, and a guide wire are supplied. For venipuncture, we use common peripheral intravenous catheters with appropriate lengths and gauge sizes through which a guide wire can be passed. In this kit, an 18-gauge needle is sufficient. In case of “Seldinger technique”-based kits, a 20- or 22-gauge needle might be sufficient because those guide wires are usually thinner than “peel-off sheath”-based kits. Further, commonly used materials such as surgical caps, masks, eye protection, sterile gloves, gowns, drapes, disinfectant sponges, gauzes, sutures with needles, scalpels, anesthetic syringes, and 1% or 2% lidocaine anesthetic solutions are also required (not shown). (**B**) **Arm position.** The patient should be asked to lie down in the supine position, which allows the upper limb to abduct, upper arm to rotate outward, forearm to supinate, and medial side of the arm to be upward for better demonstration of the basilic vein. The elbow should not be bent, and the forearm should not be pronated. (**C**) **Tips for applying the probe with the correct angle.** The probe should be applied at the correct angle.

The equipment used is shown in Fig. 3A. In the fluoroscopy room, the patient should lie down in the supine position, allowing the upper limb to abduct, upper arm to rotate outward, forearm to supinate, and medial side of the arm to be upward for better demonstration of the basilic vein. The elbow should not be bent; the forearm should not be pronated (Fig. 3B). Avascularization should be applied to the central side of the upper arm. An ultrasound device with linear-array transducers of high frequencies (5–14 MHz) should be used to scan the target vein to be punctured. A successful puncture depends on the choice of vein. The priorities for selecting a vessel are (a) adequate caliber, (b) distance from an artery, (c) not being tortuous, and (d) not too close to the elbow.

**Figure 4:**
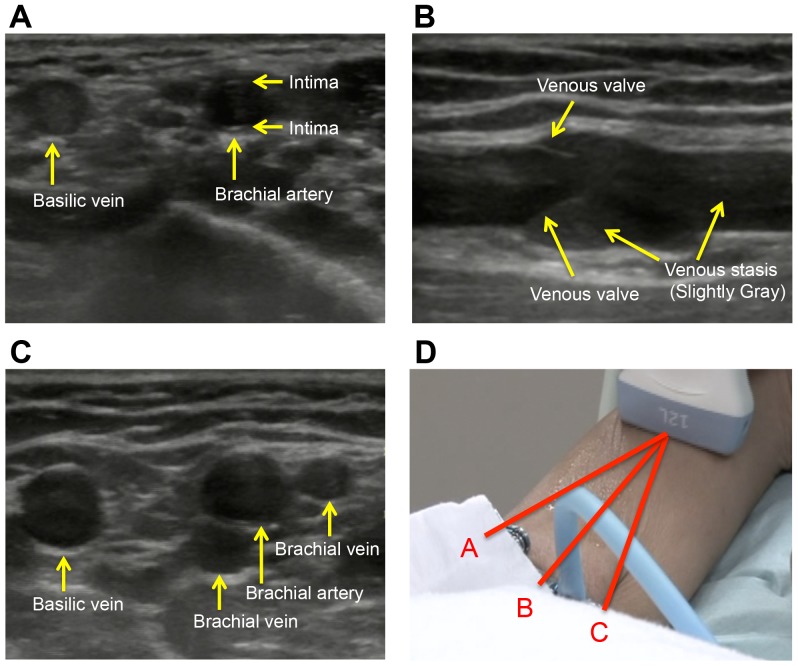
How to distinguish the artery and vein. (**A**) **Typical appearance of artery.** An artery is rounded, pulsing, and accompanied by thin high brightness of the intima beneath the adventitial circle. (**B**) **Venous stasis and valve.** Gray slightly high-echoic venous stasis is seen in the lumen and, depending on the site, in venous valves. (**C**) **Typical cross-section view of the upper arm.** A brachial artery, two accompanying brachial veins, and a basilic vein located medially far from the others are shown. (**D**) **Imaging the vessel course pattern by sweeping.** If the vein runs as B, its section will stay at the center of the monitor during sweeping. If the vein runs as A or C, it will move to the left or right on the monitor during sweeping. In this case, turn the probe orthogonal to the line (A or C). After imaging and adjustment, a 90° turn of the probe will result in a clear longitudinal view of the vein.

The probe should be applied at the correct angle (Fig. 3C); veins and arteries should be distinguished. An artery is pulsing, accompanied by thin high brightness of the intima beneath the adventitial circle (Fig. 4A) and does not collapse by compression using the probe, whereas a vein collapses (Video S1). Gray, slightly high echoic venous stasis is seen in the lumen and, depending on the site, in venous valves (Fig. 4B). Doppler mode for the ultrasound device may be useful for color visualization of arterial pulsation (Video S2).

Typically, a brachial artery, two accompanying brachial veins, and a basilic vein located medially far from them should be recognized (Fig. 4C). For practical purposes, a basilic vein is the first choice for venipuncture because it is usually solitary, fulfilling criterion (b). If it also meets criteria (a) and (c), it can be punctured. Some proximity to the elbow would be tolerated (Fig. 1). Regarding criterion (a), either equaling or surpassing that of an artery would be empirically sufficient.

To survey for criterion (c), one can recognize the vessel course pattern based on whether the venous cross section in a transverse view stays at the center (straight) or not (tortuous) by moving the probe from distal-to-proximal site (sweeping). However, if the probe is not orthogonally oriented to the directionality of the vessel, the section may move away from the center when sweeping, although the vessel is straight (Fig. 4D). Thus, several sweeps should be used to first image the orientation of the vein. Second, the probe should be applied orthogonally to the assumed long axis of the vein. Finally, a 90° turn of the probe should be made to provide a clear longitudinal view of the vein. In our method, this view is used during puncture (Video S3).

When a basilic vein puncture appears to be difficult or failed, a brachial vein meeting criteria (a) and (c) is preferred next. Appropriate real-time ultrasound guidance will enable accurate venous puncture regardless of fulfilling (b). Because a brachial vein sometimes has adequate caliber, it can be an optional puncture target. However, a median nerve occasionally runs around them, so attention must be paid for its absence in the puncture route. The cephalic vein is not chosen for puncture because its greater confluence angle to the subclavian vein often results in catheter insertion failure. If there is no optimal vein for puncture, the other arm should be surveyed.

#### Real-time ultrasound-guided venipuncture

Maximum barrier precautions are necessary. We use common peripheral intravenous catheters with appropriate lengths and gauge sizes through which a guide wire can be passed because a puncture does not always succeed in one trial (Fig. 3A).

**Figure 5:**
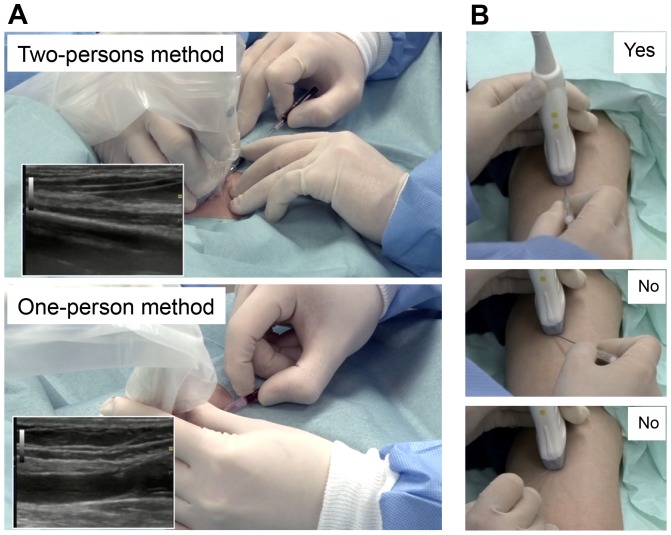
Real-time ultrasound-guided venipuncture. (**A**) **“Two-person method” and “one-person method.”** With the “two-person method,” the ultrasound-guidance step and the puncturing step are assigned separately to two operators. This can increase the success rate for beginners. (**B**) **Tips for puncture with the correct angle.** The axes between the probe and needle must be kept in one line.

There are two ways for venipuncture. We call these the “two-person method” and the “one-person method.” The former is easier for beginners. In the “two-person method,” the ultrasound-guidance and puncturing steps are assigned to two people (Fig. 5A). The operator (performing the puncture) looks only at the puncture site without seeing the ultrasound monitor and concentrates on keeping the axes between the probe and needle in one line (Fig. 5B). The assistant looks at the monitor, holds the probe to maintain a clear longitudinal view of the vein, and tells the operator where the needle tip is. After the needle is correctly introduced into the vessel, the sheath should be brought forward (Video S4), as is performed in typical peripheral intravenous replacement.

In the “one-person method,” the operator must handle the probe in one hand and the needle in the other (Video S5).

Subsequently, with this indwelling of a peripheral intravenous catheter line, the remaining procedure is nearly similar to that used for common CV port implantation.[3]-[6] The only difference is that a subcutaneous tunnel is not required.

#### Central Venous Catheter Replacement and Port Implantation

The guide wire should be inserted through the lumen of the peripheral catheter placed in the vein and carried forward until SVC is reached under X-ray fluoroscopic guidance. If there is abnormal resistance during wire passage, appropriate use of a contrast dye through the catheter may be helpful to confirm a run-through of the vessel and presence of stenosis or occlusion. After introducing the guide wire, the peripheral catheter should be withdrawn. A local anesthetic should be applied to areas about 2 cm right and left from the puncture point for a skin incision, and to areas 4 cm peripheral from these to establish a subcutaneous pocket.

**Figure 6:**
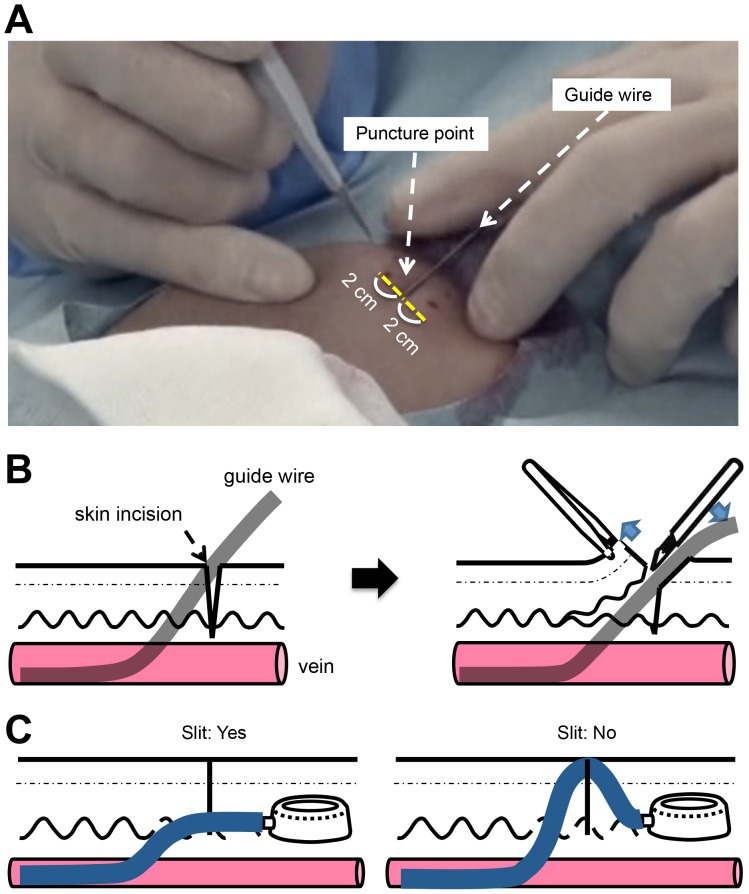
Port Implantation. (**A**) **Skin incision.** A scalpel should be used to make a skin incision from 2 cm to the right to 2 cm to the left of the puncture point. This incision should be used later as the entrance for making a subcutaneous pocket with a forceps. (**B**) **Making a slit between the skin and wire.** The connective tissues between the skin and wire should be cut with a scalpel to make a slit a few millimeters long over the wire in the puncture point. (**C**) **The purpose of a slit.** Without this step, the catheter route will be shallower from the skin surface; subsequently, the risk of catheter exteriorization will increase.

Subsequently, a scalpel should be used to make a skin incision from 2 cm to the right to 2 cm to the left of the puncture point (Fig. 6A). This incision should be used later as the entrance for making a subcutaneous pocket with a forceps. Next, the connective tissues between the skin and wire should be cut with a scalpel to make a slit a few millimeters long over the wire in the puncture point (Fig. 6B, Video S6). This has two purposes: i) to make it easier to subsequently introduce a dilator and ii) to place the catheter route deeper from the skin surface so as to reduce the risk of catheter exteriorization (Fig. 6C).

Hereafter, catheterization differs according to the kit used; one should refer to the manufacturer's manual. We describe an example of the SlimPort kit (Bard Access Systems, Salt Lake City, UT, USA) for reference. The dilator sheath should be introduced along the guide wire. After placement, the sheath should be left where it is, and the guide wire and dilator should be withdrawn. The catheter should be immediately introduced into the vessel through the sheath. The catheter should be appropriately brought to SVC. The optimal CV catheter tip location is about 2 cm passed centrally from the carina, as recognized by fluoroscopy.[7]–[10] The sheath should be peeled off.

Next, a subcutaneous pocket for a port should be made by blunt dissection using forceps (Video S7). The port and catheter should then be connected according to the manufacturer's manual. Fixing the port to connective tissue through the suture hole is optional with our upper arm method. Finally, the skin should be sutured appropriately while avoiding pricking the catheter.

## Results

### Patient Characteristics

All CV port implantations performed in our unit from January 2006 to December 2011 were included for analysis (n = 599). Only one case, in which femoral implantation was chosen because of SVC syndrome, was excluded. In total, 342 and 257 CV ports were successfully implanted in the subclavian area and the upper arm, respectively. Patient characteristics are shown in Table 1. No significant differences in gender ratios and mean ages were observed between the groups. Both groups had similar patient characteristics except for the colorectal cancer population.

### Procedural Success and Periprocedural Complications

Procedural success and complications are shown in Table 2. No significant difference in the procedural success rate was observed between the subclavian and upper arm groups (99.42% vs. 98.83%; P = 0.66). Although the implantation site had to be changed in a few cases, all procedures were eventually successful by switching the site between the subclavian area and the upper arm. No fatal outcomes due to complications were observed in either group. There was no periprocedural complication, including pneumothorax or arterial puncture, in the upper arm group, although the differences were not significant. This was possibly due to the very low number of patients in the subclavian group who developed the periprocedural complication and the experience of the attending physicians in placing ports via ultrasound guidance (discussed later).

### Postprocedural Complications

Although this was a retrospective single-center analysis, the over-all postprocedural complication rates were significantly lower in the upper arm group (Table 2). This difference can be explained because mechanical complications, such as catheter occlusion, fracture, and malposition, were unlikely to occur in the upper arm group for reasons described in the Discussion section (i.e., no “pinch-off” point or steep turning section in the catheter line). The incidence of catheter occlusion was also significantly lower in the upper arm group. The incidences of other major complications, such as infection or venous thrombosis, were equivalent. All infections were diagnosed during long-term follow-up, and no procedure-related early (within 1 week after the procedure) infections were observed in either group. Thus, CV port implantation in the upper arm had equivalent safety and advantages compared with subclavian implantation in our cohort of patients (Table 2).

## Discussion

### Periprocedural Advantages of Upper Arm Implantation

Critical procedural complications during subclavian implantation are pneumothorax and arterial puncture.[6],[11] In the present retrospective study, the difference between the incidences of these two periprocedural complications was not significant, although no such complications occurred in patients in the upper arm group (Table 1). This seems to be attributable to lower prevalence of these complications in our subclavian group (pneumothorax, 0.88%; arterial puncture, 0.58%; total, 1.46%) than in the subclavian group in other reports in the literature (pneumothorax, 1.5%–3.1%; arterial puncture, 3.1%–4.9%; total, 6.2%–10.7%).[6] We attribute the lack of complications to the fact that we routinely used ultrasound in the subclavian group and the operators were the same physicians who expertly accomplished upper arm venipuncture with ultrasound, which is usually more difficult than subclavian implantation because of the diameter of the vein. In other words, no matter how skillful the operator is in ultrasound guidance, accidental periprocedural complications, such as pneumothorax, cannot be eliminated completely in subclavian implantation unless the risk is structurally excluded. In this regard, upper arm CV port implantation has several physical advantages.

First, because of anatomical reasons, pneumothorax does not occur in upper arm implantation. Second, unlike subclavian/internal jugular/femoral puncture, no landmark method for the upper arm exists, forcing an operator to use ultrasound, eliminating arterial puncture risk. Third, even if arterial puncture occurs, bleeding can be easily stopped by hand pressure on the puncture site, similar to the procedure used after an arterial blood gas test. Fourth, avascularization, applicable only in this procedure, can reveal venous stasis by its discriminative slightly gray color in the ultrasound view and subsequent vessel enlargement facilitates venipuncture. Finally, unlike other sites, the solitary basilic vein runs apart from nearby arteries, circumventing accidental arterial cannulation that causes serious complications.[12]–[16] Therefore, this procedure can be performed safely by trained physicians and is not limited to surgeons. Despite being medical oncologists, we have successfully implanted over 400 upper arm CV ports without serious complications during the last 7 years.

### Postprocedural Advantages of Upper Arm Implantation

**Figure 7:**
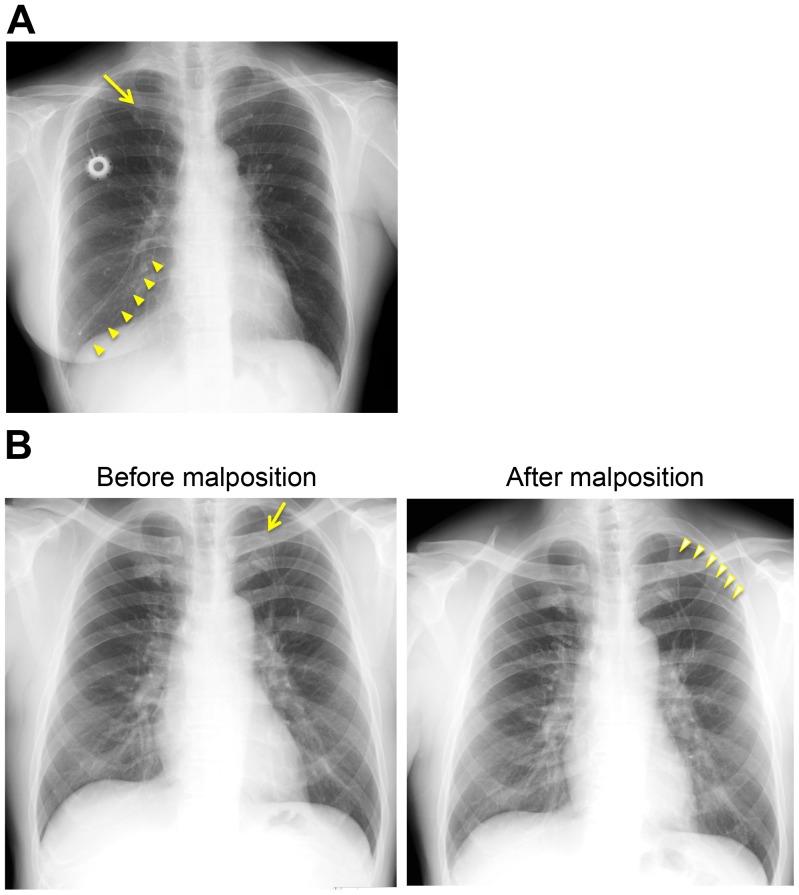
Images of postprocedural complications that could be prevented with an upper arm CV port. (**A**) **Catheter pinch-off syndrome and fracture.** *Arrow* indicates a “pinched-off” and fractured site of a catheter. *Arrowheads* indicate the fractured distal catheter fragments that had migrated into the pulmonary artery through the heart. (**B**) **Catheter inversion.** Left panel, *Arrow* indicates a normal catheter placed centrally. Note that there is a sweep turning point at this puncture site that may cause tension derived from an elastic restoring force. Right panel, *Arrowheads* indicate the peripherally inverted distal portion of a catheter for the same case.

The overall postprocedural complication rate was significantly lower in the upper arm group (Table 2). “Pinch-off syndrome” (Fig. 7A), occurring in approximately 1% subclavian procedures,[6], [17]–[21] does not occur during upper arm implantation because of anatomical reasons. Distal catheter migration from the puncture point (Fig. 7B) is also unlikely to occur in the straightforward upper arm CV lines because there are no steep turning sections causing tension from an elastic restoring force. Such parts are usually observed in subclavian or internal jugular procedures.[22]–[24]

### Possible Advantages of Upper Arm Implantation

In terms of other plausible advantages, which have yet to be elucidated objectively by inquiry using scales, a patient's fear of an upper arm puncture might be less than a subclavian or internal jugular puncture. It generally takes less effort to roll up a sleeve to provide access to the upper arm port than to completely remove a shirt for access to the subclavian port (Fig. 1). Upper arm implantation does not leave scars on the neck or chest, which may prevent patients from wearing wide-open neck clothes because of cosmetic concerns.

Further, no significant difference in the frequency of infection or thrombosis between subclavian and upper arm procedures has been suggested.[25]–[36] Patient skin temperatures and skin aerobic/anaerobic flora density were significantly lower on the forearm than on the subclavian area,[37] probably impacting adverse infection incidence.

### Possible Limitations and Solutions

There are two possible limitations of the procedure. The operator should be skillful in performing venipuncture under real-time ultrasound guidance. However, training of appropriate staff, as has been performed in our division, can address this problem. This report and videos may provide useful training guidance. Further, a patient has to take the needle out of a port with one hand. This can be enabled using a needle with a mechanism that can enclose the needlepoint by single-hand manipulation (e.g., HuberPlus; Bard Access Systems).

### Conclusions

As more physicians become capable of performing upper arm CV port placement, more patients will receive benefits, such as elimination of the pain of routine peripheral intravenous access, infusional angialgia, and extravasation of cytotoxic agents during chemotherapy. Several skilled operators may also contribute to minimizing the need for busy surgeons to perform routine CV port implantation procedures. The procedure is also useful for in-home care or palliative medicine; an example would be a venous line for total parenteral nutrition or opioids. Wider use of CV port implantation should reduce medical costs by shifting in-hospital care to outpatient care (continuous infusion chemotherapy) and in-home care (palliative medicine).

Particularly in Japan, the recent social demands for outpatient cancer chemotherapy, in-home care, and palliative care have been rapidly increasing because of low birth rate and increased longevity, the universal health insurance system since 1961, and other reasons. Thus, CV ports can be used for multiple functions and can contribute to secure, safe, and seamless oncological care from anti-cancer therapy to palliative medicine.

We propose that upper arm CV port implantation, compared with subclavian implantation, can provide safety and comfort benefits to both medical professionals and cancer patients. We hope that this procedure will become more common and eventually be validated in prospective multicenter randomized clinical trials regarding its non-inferiority or superiority to other subclavian or internal jugular procedures with respect to safety, maintenance of quality of life, and cost-effectiveness. In fact, we are planning a study to directly address such issues regarding other possible advantages as well as those identified in the present study.

## Supporting Information

Video S1 **Compressibility by probe.** An artery does not collapse by compression using the probe, whereas a vein does collapse.
(MOV)

Video S2 **Color visualization of arterial pulsation in a Doppler mode.** If necessary, a Doppler mode for the ultrasound device is useful to generate color visualization of arterial pulsation.
(MOV)

Video S3 **Longitudinal view of the vein.** The probe should be applied orthogonally to the assumed long axis of the vein. Next, a 90° turn of the probe should be made to provide a clear longitudinal view of the vein. In our method, this view is used during puncture.
(MOV)

Video S4 **“Two-person method.”** Ultrasound guidance by the assistant enables the operator to focus solely on puncturing, as is performed in routine peripheral intravenous placement. After the needle is correctly introduced into the blood vessel, it should be placed down and the sheath should be brought forward.
(MOV)

Video S5 **“One-person method.”** In the “one-person method,” the operator has to handle the probe in one hand and the needle in the other.
(MOV)

Video S6 **Making a slit between the skin and wire.** The connective tissues between the skin and wire should be cut with a scalpel to make a slit a few millimeters long over the wire in the puncture point. Without this step, the catheter route will be shallower from the skin surface, and subsequently, the risk of catheter exteriorization will increase.
(MOV)

Video S7 **Making a subcutaneous pocket.** A subcutaneous pocket should be made by blunt dissection using forceps.
(MOV)
